# A Time-Differential BOCDA Sensor Measurement System Applied to a 1 km Long SMF Using a Semiconductor Optical Amplifier as a Pump Chopper

**DOI:** 10.3390/s24082417

**Published:** 2024-04-10

**Authors:** Bo-Hun Choi

**Affiliations:** Department of Semiconductors, Dong-A University, Saha-gu, Busan 49315, Republic of Korea; adamchoi@dau.ac.kr

**Keywords:** residual strain, optical fiber, Brillouin scattering, strain sensor, semiconductor amplifier, EDFA

## Abstract

A time-differential (TD) Brillouin optical correlation domain analysis (BOCDA) sensor system was applied to measure the Brillouin gain spectrum of a 1 km long sensing optical fiber. The optical delay line used in all BOCDA measurement systems was eliminated in the TD-BOCDA system by using a bit-delayed modulation relationship between the probe and pump lightwaves. These lightwaves were phase modulated using 2^16^-1 pseudo-random binary sequence codes at 5 Gbps. A 2 cm dispersion-shifted fiber placed at the end of the 1 km optical fiber was distinctly identified by the Brillouin frequency extracted from the Brillouin gain spectrum measurement. To investigate the measurement stability of the TD-BOCDA system, experiments were conducted under two different pumping conditions. A semiconductor optical amplifier (SOA) and an intensity modulator (MOD) were compared for the pump chopper used in the TD-BOCDA system to detect the extinction ratio of the pump and the resulting noise in the Brillouin gain measurement. The stability of the Brillouin frequency measurement from the Brillouin gain spectrum in the TD-BOCDA system was investigated by increasing the average value of the measurement using either the SOA or MOD. The repeated-measurement deviation of the system with the SOA was only half of the deviation observed in the system with the MOD. The performance of TD-BOCDA is equivalent to or better than that of conventional BOCDAs in terms of measurement reliability. Moreover, TD-BOCDA is free from the drawbacks of traditional BOCDA, which uses time-delayed fibers and varies the bit rates.

## 1. Introduction

Brillouin optical correlation domain analysis (BOCDA) sensing technology has been proposed using the Brillouin scattering effect in optical fibers and has been widely applied in structural health-monitoring systems [1,2]. This sensing technology enables the measurement of distributed strain and temperature variations in optical fibers [3,4,5]. In BOCDA technology, continuous probe and pump lightwaves propagate in opposite directions within a closed fiber loop. When the frequency difference between two lightwaves becomes the inherent Brillouin frequency of the optical fiber, the probe experiences gain transfer from the pump, a process known as stimulated Brillouin scattering (SBS). SBS occurs at specific locations in the fiber depending on the correlation between the two waves. This correlation can be adjusted by applying a multi-gigahertz pseudo-random binary sequence (PRBS) signal to either a frequency- or phase-modulated probe and pump [6,7,8].

The BOCDA sensor system, which employs the phase modulation technique, has successfully achieved sensing distances of several hundred meters while maintaining a sub-centimeter spatial resolution. Notably, this BOCDA technology eliminates the tradeoff between long sensing distances and fine spatial resolutions, a limitation observed in its competitor, the Brillouin optical time domain analysis (BOTDA) technology [8,9,10].

However, BOCDA technology requires an optical delay line in a closed loop to position the sensing fiber in the proper correlation region arising from the interaction of two lightwaves [1,5,8,9,10]. The length of the optical delay line depends on the length or spatial resolution of the sensing fiber. The optical delay line of the BOCDA measurement system must be replaced whenever its length and resolution conditions change. The delay line is typically several to tens of kilometers long. Furthermore, temperature changes affect the accuracy of the detected position when such long delay fibers are used [11]. Therefore, we propose a novel method called time-differential (TD) BOCDA, which utilizes two PRBSs that are identical except for their bit-delayed relation [12]. The TD-BOCDA system has been demonstrated to not only eliminate the necessity for an optical delay line, but also to easily determine the absolute position on the sensing fiber without requiring reference measurements to establish the relationship between the location control parameters and the actual fiber position, which is essential in a BOCDA system. However, the PRBS generator (Tektronix, AWG70000A) for two channels with a bit-delay relation has a slow computer interface speed, making it difficult to measure every location on a sensing fiber longer than 100 m. More importantly, there has been no analysis of the measurement stability of the TD-BOCDA system.

In this paper, we applied this novel method to a sensing fiber with a length of 1 km that included several different types of optical fibers. A 5 Gbps PRBS code in the TD-BOCDA system was used for a spatial resolution of 2 cm over the entire sensing length. Lock-in amplifiers (LIAs) with pump choppers are commonly used to amplify inherently weak SBS gains in BOCDA systems and detect small probe gains. In this experiment, a MOD and an SOA were compared as pump choppers to quantitatively validate the measurement stability of the TD-BOCDA system. The measurement deviations in detecting the Brillouin frequency, which indicates changes in strain or temperature, were measured in the TD-BOCDA sensor systems using these two pump choppers.

## 2. Experiment Setup and Operating Principle for the Fiber Optic Sensor Measurement System

**Figure 1:**
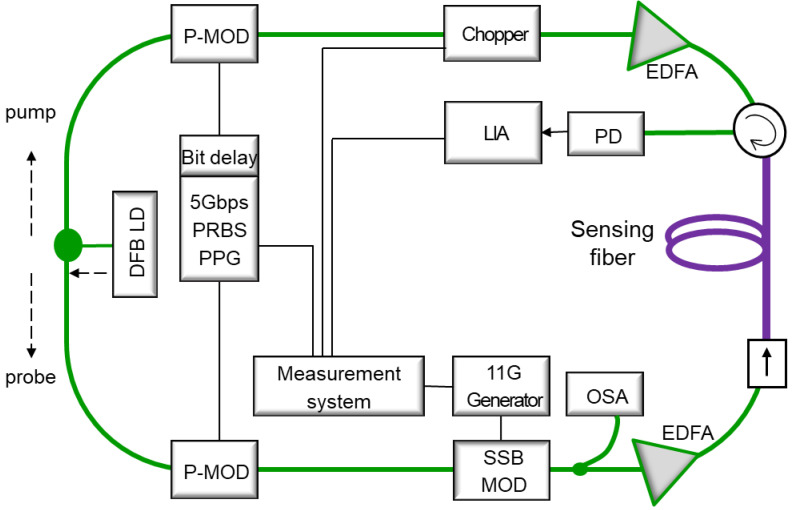
Experimental setup for the time-differential Brillouin optical correlation domain analysis (TD-BOCDA) sensor measurement system; P-MOD (phase modulator), LIA (lock-in amplifier) PD (photodetector), SSB (single sideband), PPG (pulse pattern generator).

The experimental setup for the TD-BOCDA sensor measurement system is shown in Figure 1. A continuous lightwave from a distributed feedback (DFB) laser diode was split by a 3 dB coupler into two opposite directions as the probe and pump lightwaves. They were optically modulated by their respective phase modulators, which were driven by 2^16^-1 bits of PRBS from a pulse pattern generator (PPG) of 5 Gbps. The bit length of the 2^16^-1 PRBS code was 65535, which could accommodate up to 1310 m of the sensing optical fiber with a spatial resolution of 2 cm. The same PRBS codes were applied to the two modulators; however, they had a bit delay relation [12]. After the phase modulation, the probe was again modulated by a single-sideband (SSB) modulator, which caused the carrier frequency to downshift from the pump lightwave by ~11 GHz. The SBS in the sensing optical fiber was generated by the frequency difference between the probe and pump lightwaves. After amplification using an Er-doped fiber amplifier (EDFA), the probe was introduced into the sensing fiber through an optical isolator. The pump sequentially passed through a phase modulator driven by PRBS codes and an optical chopper modulated by a sine wave of approximately 3 MHz. A chopper was required for the LIA to be used for optical signal detection. After chopping, the pump was amplified by a high-power EDFA of 27 dBm and inserted into a sensing fiber through an optical circulator. The circulator and isolator positioned at both ends of the sensing optical fiber prevented the lightwaves from propagating further and restricted the occurrence of the SBS effect to only the sensing optical fiber. After the isolator, the probe was amplified on the sensing fiber by the pump traveling in the opposite direction of the fiber and then received by the detector through the circulator. Figure 1 also shows the connection of the measurement control system, where the PPG, bit delay, SSB modulation, pump-chopping, and LIA are interconnected and controlled together, resulting in the collection of SBS.

**Figure 2:**
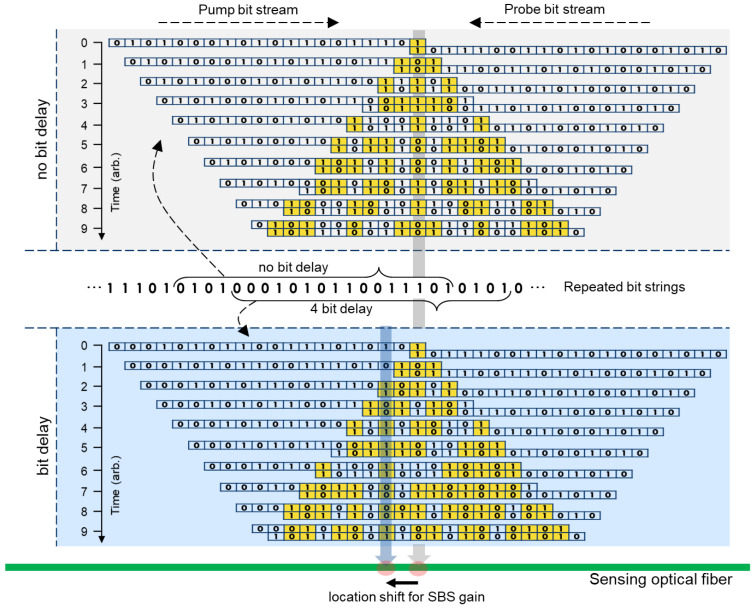
Operating principle of the TD-BOCDA measurement system.

The operating principle of TD-BOCDA is conceptually illustrated in Figure 2, which explains how the location on the sensing fiber where the SBS gain is measured is determined in this measurement system. The long line at the bottom indicates the sensing fiber. On top of this, the time-dependent interaction between the two optical signals is shown separately for the case with no bit delay between the two signals and the case with a 4-bit delay. First, let us consider the case with no bit delay. Two optical signals, the pump and probe, phase-modulated by 0 and 1, are depicted traversing the sensing fiber in opposite directions. The signals consist of 20 bits each for the sake of the example, and because they were generated by the same PRBS generator, they have mirror-symmetric bit strings. They first meet at the midpoint of the sensing fiber, which is at the same distance from both modulators. The moment when the first bit overlaps is denoted as t = 0 in the figure. As time passes, the signals increasingly overlap, and the phases of the two overlapping signals may or may not match. However, there is only one location on the fiber where the two bits are always in phase, irrespective of time. (The experimental condition for this is that the bit stream must be longer than the length of the fiber, which is why a stream length of 2^16^-1 was used in the experiment.) This location is at the same distance from both modulators, where the two optical signals are always in phase, thus maintaining the correlation between the two signals and resulting in an SBS gain. Outside this location, it is possible for the two signals to match in phase for a short period; however, they cannot remain in phase long enough to generate an SBS gain. (Bits with matching phases are distinguished in different colors, as shown in Figure 2.) Therefore, the SBS always occurs at a specific location on the sensing fiber, and the spatial length of this location is the minimum measurement interval corresponding to a time length of one bit. This is called the spatial resolution of the measurement system. An optical signal modulated at 5 Gbps provides a spatial resolution of 2 cm.

In the conventional BOCDA schemes, two hardware devices are required to change the location where the SBS gain is generated. First, you need to change the bit rate, and therefore, it adjusts the time interval of one bit. Second, you need to use an optical fiber for a time delay of several kilometers or tens of kilometers [9,13,14]. However, TD-BOCDA eliminates the need for time-delayed optical fiber and the need for complex adjustments to the bit rate of the phase modulator. This novel method simply requires a time delay between the exact same bit streams applied to the two phase modulators with the amount of time delay determined by the number of delayed bits. The middle of Figure 2 shows the repeating bit strings and how the bit combinations are chosen for no-bit delay and 4-bit delay. This bit delay is achieved by adjusting the generation sequence of one set of bit string input into the PRBS device. The overlap of the two optical signals in the case of 4-bit delay is also included in Figure 2, which is given just above the sensing fiber line. The location of the fiber where the two optical signals meet in a mirror image shifts from its location when there is no bit delay to a distance equal to the number of bits delayed. Thus, controlling the number of delayed bits enables the control of the location where the SBS gain occurs. A two-channel PRBS generator for the bit delay was custom-built for the experiments. The generator was connected to the measurement system together with an SSB modulator and receiver and operated by an automatic measurement program. The location of the SBS gain is shown on the sensing fiber in Figure 2, illustrating that this location moves in proportion to the bit delay.

**Figure 3:**
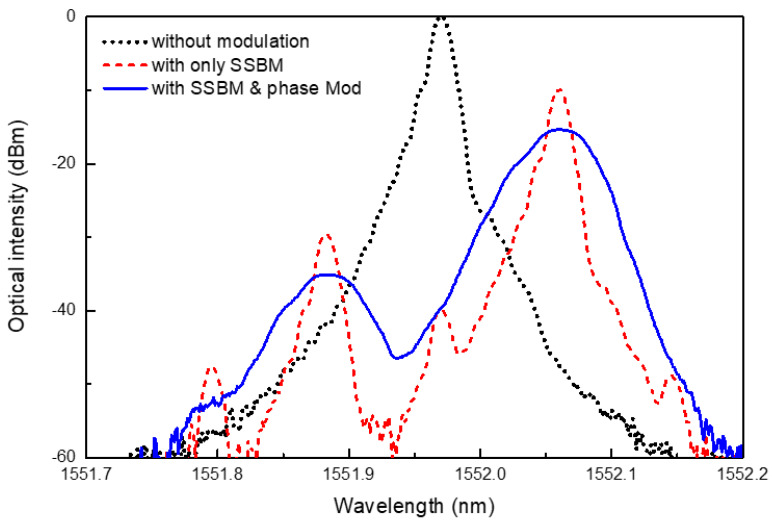
Optical spectrum of the probe lightwave in the TD-BOCDA with no modulation, with only single-sideband modulation (SSBM), and with SSBM and phase modulation.

The optical spectra of the probe lightwaves are shown in Figure 3. The lightwaves were measured using a 1% tap after passing through the SSB modulator; this measurement point was marked as an optical spectrum analyzer (OSA), as shown in Figure 1. The spectrum without both phase modulation and SSB modulation is represented by a dotted line with a peak wavelength of 1551.97 nm. When SSB modulation was applied, the spectrum changed, as indicated by the dashed red line. The center wavelength of the 1551.97 nm was completely suppressed, and the peak wavelength shifted by 0.09 nm (11 GHz) to 1552.06 nm. The side-mode suppression ratio of this spectrum exceeded 20 dB. A wavelength difference between the probe with SSB modulation and the pump without SSB modulation was required to induce the SBS effect in the sensing fiber. The spectrum changed again when phase modulation was applied in conjunction with SSB modulation. This is indicated by the solid blue line in the figure, which clearly demonstrates the linewidth broadening of the spectrum owing to the modulation chirping of the 5 Gbps PRBS code.

## 3. The Novel TD-BOCDA Measurement System Applied to a Sensing Optical Fiber of 1 km Length

**Figure 4:**
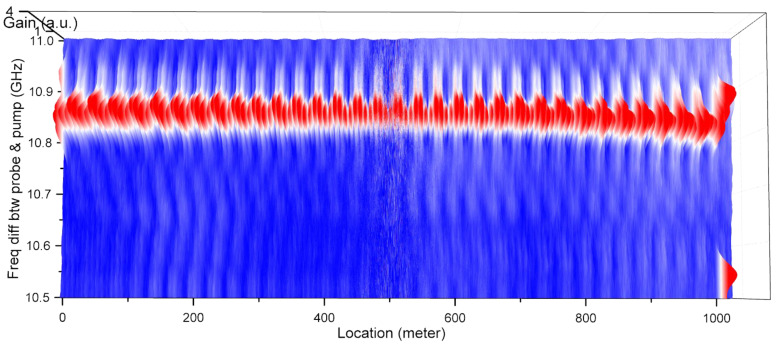
Brillouin gain spectra measured at all locations over 1 km.

The TD-BOCDA sensor system was applied to a standard optical single-mode fiber (SMF) with a length of 1 km as the sensing fiber. Figure 4 shows the three-dimensional Brillouin gain spectra measured at all the locations. At each measurement location, the frequency difference between the pump and probe was swept between 10.5 and 11 GHz with a resolution of 0.1 MHz, and the Brillouin gain spectrum was measured. The peak frequency difference for the highest gain at each fiber location, known as the Brillouin frequency, was determined from this spectrum. Each optical fiber possesses an inherent Brillouin frequency, whose value shifts with changes in strain or temperature. For example, the Brillouin frequencies of an SMF and a dispersion-shifted fiber (DSF) are around 10.8 GHz and 10.5 GHz, respectively [5]. To evaluate the accuracy of the 2 cm spatial resolution over a 1 km SMF, a 2 cm length of DSF was spliced at the exit end of a 1 km long SMF toward the pump input. Almost all the Brillouin frequencies of the SMF occurred near 10.85 GHz. However, the Brillouin frequency shifted slightly toward shorter wavelengths with increasing distance, owing to the position-dependent tension that occurs while winding a kilometer-long fiber spool. It is evident that there was a single Brillouin frequency peak of 10.53 GHz for the 2 cm DSF at the 1 km location. The Brillouin frequency shifts at the initial and final locations were distinctly measured and differed from the Brillouin frequencies measured at the most intermediate locations. These shifts were due to the different types of SMFs used.

**Figure 5:**
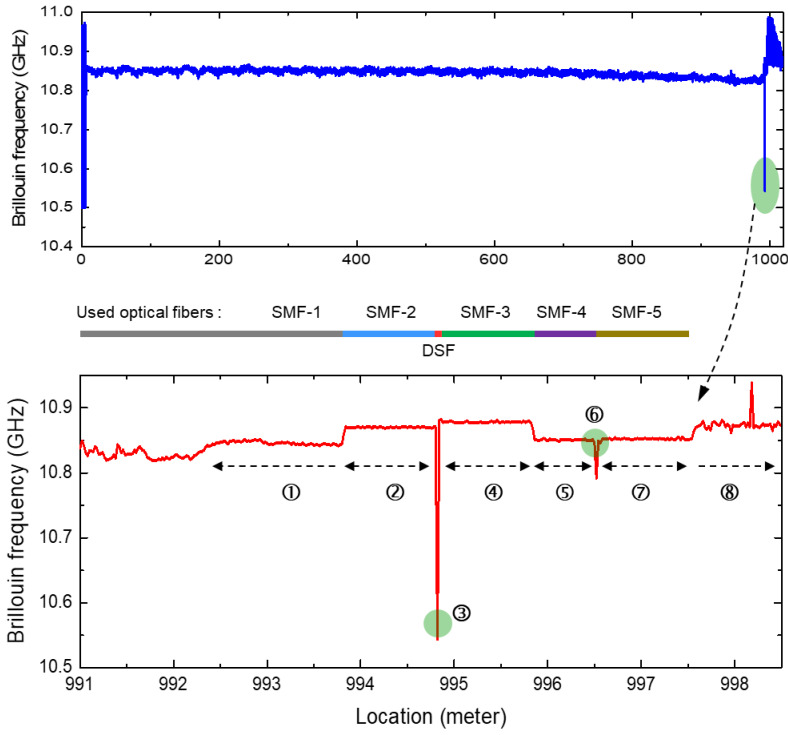
Brillouin frequencies extracted from Brillouin gain spectra at all 1 km locations in the **top** graph and at the end area of 1 km locations including a 2 cm long DSF in the **bottom** graph. The diagram in the middle of the two graphs shows the optical fiber configuration at the 1 km end.

**Figure 6:**
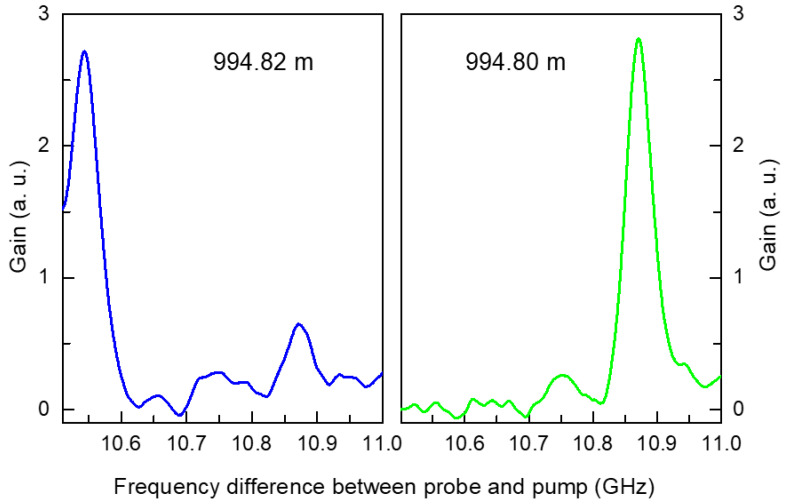
Brillouin frequency spectra measured at 994.80 m (**right**) and 994.82 m (**left**).

Figure 5 presents the Brillouin frequencies extracted from the gain spectra in Figure 4. Extracting the precise Brillouin frequency from this spectrum is critical to the accuracy of this measurement system. Both hardware approaches employing a probe or pump with multiple frequencies and software approaches involving the mathematical curve fitting of the extracted spectrum have been applied to enhance the accuracy [15]. In this paper, to clearly demonstrate the advantages of the proposed method, curve fitting was not applied. For reference, spectral measurements in BOCDA systems have been performed by our research group over the years under various conditions, and Lorentzian and Gaussian curve fittings have been applied. Depending on the shape of the spectrum, in some cases, the fitting caused more errors in the frequency compared to peak extraction; in other cases, it made no difference. The top graph in Figure 5 shows that almost all the Brillouin frequencies were approximately 10.85 GHz because of the one-type SMF with a length of 1 km. Both end regions exhibited significant frequency fluctuations, which were attributed to noise measured in the area outside the sensing fiber. At the end of the 1 km distance, a discrete location is marked with a green ellipse, which indicates a frequency of 10.53 GHz. This corresponded to the location of a 2 cm long DSF, and among the 51,000 measurement points measured with a spatial resolution of 2 cm, this point (③) was successfully detected. The bottom graph shows an enlarged view of this end area (including the DSF), which is marked from ① to ⑧ according to the Brillouin frequency shift. The bold line between the top and bottom graphs indicates the different types of optical fibers used according to the location. A 1 km SMF spool was used up to area ① from the start point, and this area, in particular, was the unwound end of the spool. For areas ② and ④, two types of 1 m long SMFs from Samsung Electronics and Corning Inc., which were spliced to both ends of the DSF (③), were used. For areas ⑤ and ⑦, the fibers were attached to the fiber connectors, one to the sensing fiber, and the other to the optical isolator. These two connectors were connected by a fiber adaptor, and the change in tension produced by this adaptor was also measured as the change in the Brillouin frequency at ⑥. The Brillouin frequency at the adaptor location shifted to a lower frequency, and this meant fiber contraction at the measurement point. In area ⑧, the Brillouin frequencies could not be detected and only exhibited noise because of the absence of a sensing fiber. This is evident from the Brillouin gain spectra shown in Figure 4. In Figure 5, the most dramatic change in Brillouin frequency is near 994 m. The Brillouin frequency spectra measured around this location are presented in Figure 6. The right graph was measured at 994.80 m and the left graph at 994.82 m, 2 cm beyond this. The frequency difference between the probe and pump was swept from 10.5 GHz to 11 GHz once for each spectrum measurement, and the time taken for each sweep was 0.1 s. For the entire fiber length, there were more than 50,000 measurement points in total, so the measurements in Figure 4 for all spectra took about an hour and a half.

**Figure 7:**
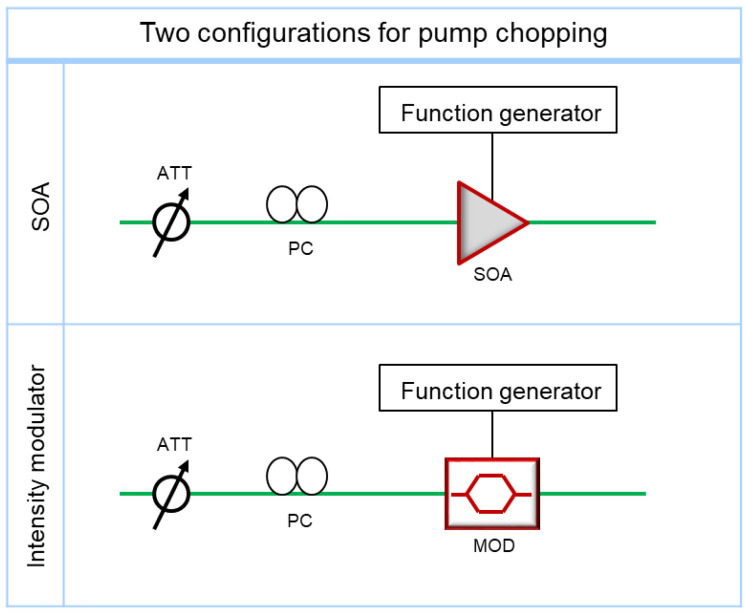
Experimental setups for two configurations using an SOA or intensity modulator for pump-chopping in the TD-BOCDA measurement system.

The measurement stability of the Brillouin frequency is one of the most important performances of a Brillouin sensor measurement system. The stability of the TD-BOCDA system was investigated, and experiments were conducted under two different pump light conditions. In the experiment of Figure 4, a semiconductor optical amplifier (SOA) was used as the pump chopper. Intensity lithium niobate modulators have been used as choppers in previous experiments, including ours [7,8]. Therefore, these two devices were compared in the TD-BOCDA sensor measurement system to detect the Brillouin scattering gain; their experimental configurations are illustrated in Figure 7. Before entering the chopper, the input power was regulated using a variable attenuator and a polarization controller for optimal input power adjustment. The chopper was operated using a function generator with a ~3 MHz sine function. The peak-to-peak and offset voltages for the electrical signals applied to the two comparison devices were found to be sensitive to the Brillouin gain and were optimized for the best performance in each configuration.

**Figure 8:**
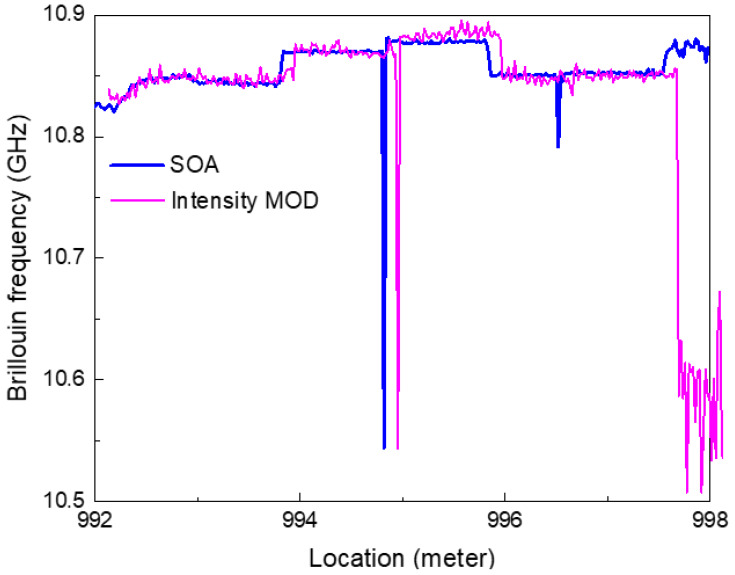
Brillouin frequencies measured at the end area of the 1 km long optical fiber, including a 2 cm long DSF, using a semiconductor amplifier (blue line) and an intensity modulator (red line). The red line was intentionally shifted by 10 cm along the horizontal axis for visual comparison with the blue line.

The Brillouin gain spectrum and Brillouin frequency were obtained when a MOD was applied to the TD-BOCDA sensor system. The results were compared with measurements obtained using an SOA. Figure 8 shows the Brillouin frequencies at the end area of 1 km in length, including the 2 cm DSF. Both measurements clearly indicate the location of the 2 cm DSF, and the two graphs are consistent with the detection location. The frequency graph with a MOD was intentionally shifted by 10 cm about the horizontal axis, representing the location for visual comparison with the graph with SOA. Both measurements accurately captured the locations of all different types of optical fibers. However, the measurement system employing a MOD exhibited relatively large frequency fluctuations in each fiber area.

**Figure 9:**
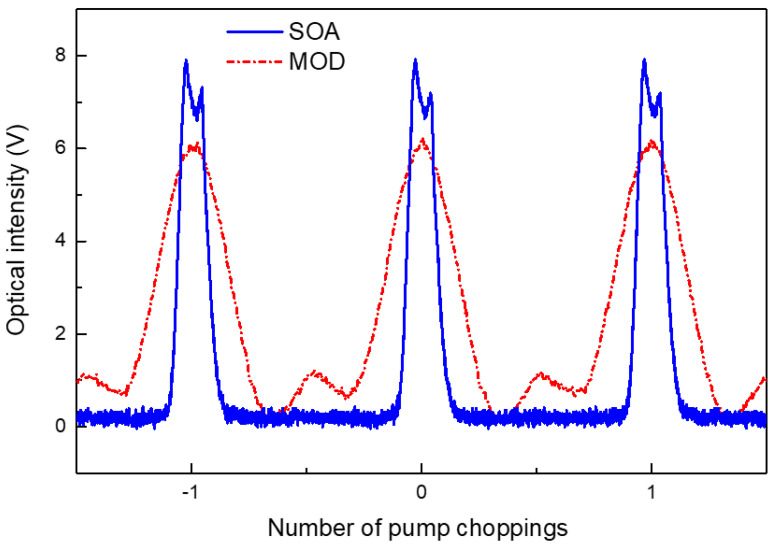
Time responses of the chopped pumps using an intensity modulator or SOA for comparison. The horizontal and vertical axes are in arbitrary time and relative voltage units, respectively.

The time response of the chopped pump amplified by the high-output-power EDFA was analyzed using an oscilloscope. The output from the EDFA was attenuated by 20 dB and fed into a photodetector with a bandwidth of 125 MHz. Figure 9 shows the time responses of the chopped pumps using the MOD and SOA for comparison. The horizontal and vertical axes represent arbitrary time and relative voltage units, respectively. Although a sine function was applied to the choppers, the combination of a chopper and EDFA distorted the time response of the pump output. The MOD performed better in terms of the pump-on and pump-off duty ratios, whereas the SOA achieved a higher extinction ratio.

**Figure 10:**
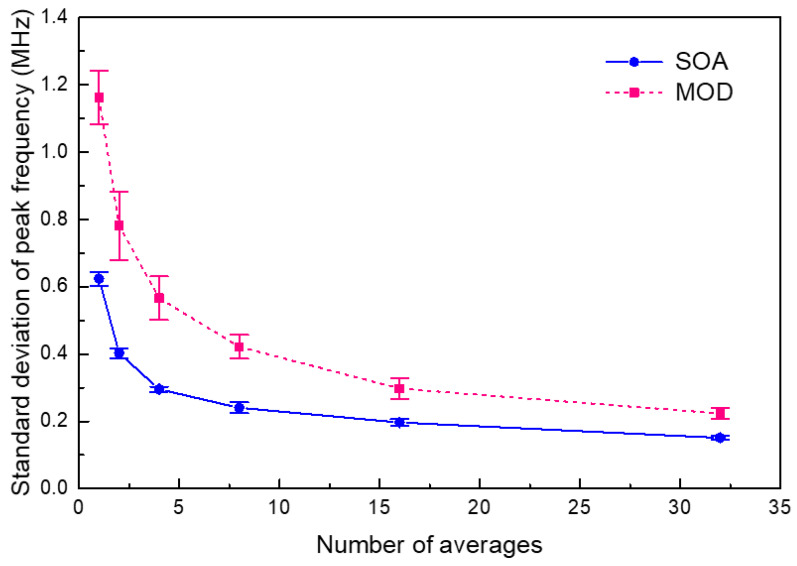
Standard deviations of the Brillouin frequency measurements in the TD-BOCDA sensor system using an SOA or intensity modulator as a pump chopper, as the number of averaged measurements increases from 1 to 32.

The stabilization of the Brillouin frequency measurement was evaluated for the TD-BOCDA sensor system using either an SOA or a MOD as the pump chopper, as shown in Figure 10. At a specific location of 997 m at the end of a 1 km distance, the Brillouin frequency detection was averaged over 1, 2, 4, 8, 16, and 32 times, and this averaged detection process was repeated 50 times. Figure 10 presents the standard deviations of the repeated averaged measurements. In the case of an SOA, the deviation was 0.62 MHz over fifty repeated measurements without the detection average. When the number of averages increased to 32, the standard deviation reduced to 0.15 MHz. This value corresponded to a strain of 3 με, considering a strain coefficient of 20 με/MHz [16]. When averaged over 32 runs, the Brillouin frequency deviation with the MOD increased by 50% compared with that with the SOA. The deviation increased by up to 100% compared with the case with the SOA without averaging. This suggests that using an SOA yields more accurate results for measuring the Brillouin frequency than using a MOD. The reason for this is estimated to be the better extinction ratio between pump-on/off compared with the case with an SOA. Compared with other Brillouin optical sensing measurement systems, the BOTDA method, which is a competing technology of BOCDA, exhibited a deviation of ~2 MHz [17], while traditional BOCDA methods have shown deviations in the range of 1~2 MHz [7,10,18]. As shown in Figure 10, under conditions without averaging, the experiment employing a MOD for pump chirping exhibited a deviation of 1.18 MHz, whereas that using an SOA exhibited a deviation of 0.62 MHz. This result demonstrates that the performance of TD-BOCDA is equivalent to or better than that of conventional BOCDAs in terms of measurement reliability. Simultaneously, TD-BOCDA is free from the drawbacks of conventional BOCDAs, such as the use of time-delayed optical fibers and varying bit rates.

## 4. Conclusions

The Brillouin gain spectra of 1 km long sensing optical fibers were measured using a novel TD-BOCDA sensor measurement system. The optical delay line used in all the BOCDA measurement systems was replaced by the bit delay relationship between the probe and pump lightwaves in the TD-BOCDA system. These lightwaves were phase-modulated using a 5 Gbps PRBS code of 2^16^-1 bit streams. The 1 km long fiber comprised six types of optical fibers, each featuring a different inherent Brillouin frequency, including a 2 cm dispersion-shifted fiber. They were clearly distinguished with a spatial resolution of 2 cm in the Brillouin gain spectrum measurements. The SOA and the intensity modulator were compared in the TD-BOCDA sensor system as a pump chopper. The pump lightwave with the SOA showed a better extinction ratio between the pump-on and pump-off states, resulting in less measurement noise when detecting the Brillouin frequency extracted from the Brillouin gain spectrum. The measurement stabilization of the Brillouin frequency in the TD-BOCDA system using an SOA or an intensity modulator was investigated as the number of averaged measurements increased from 1 to 32. As the number of averages increased to 32, the standard deviation decreased up to 0.15 MHz, which corresponded to a strain of 3 με. The standard deviation of the measurement of the TD-BOCDA system using the SOA was only half that of the system using an intensity modulator.

## Data Availability

Data are contained within the article.
